# Editorial: The global threat of carbapenem-resistant gram-negative bacteria, volume II

**DOI:** 10.3389/fcimb.2023.1196488

**Published:** 2023-04-26

**Authors:** Ziad Daoud, Milena Dropa

**Affiliations:** ^1^ College of Medicine, Central Michigan University, Mount Pleasant, MI, United States; ^2^ Faculty of Public Health, University of São Paulo, São Paulo, Brazil

**Keywords:** carbapenems resistance, gram-negative (G -) bacteria, mechanisms of resistance, KPC carbapenemase, Escerichia coli, Klebsiella pneumoniae

Multidrug-resistant bacteria pose a significant threat to global public health (World Health Organization, 2017; Centers for Disease Control and Prevention, 2020). In this context, many gram-negative bacteria have developed resistance to carbapenem antibiotics, which are often considered the last resort for treating severe infections caused by these organisms (Magiorakos et al., 2012; Tacconelli et al., 2017; Van Duin and Doi, 2017). Infections caused by carbapenem-resistant bacteria are associated with higher mortality rates and longer hospital stays, leading to increased healthcare costs. The emergence and spread of carbapenem-resistant bacteria are driven by several factors, including the overuse and misuse of antibiotics, inadequate infection control measures, and the global movement of people and goods. Addressing the threat of carbapenem-resistant gram-negative bacteria requires a multifaceted approach that includes increased surveillance, prudent antibiotic use, improved infection control measures, and the development of new antibiotics and alternative therapies.

The 11 manuscripts in this research issue revolve around the topic of resistance to carbapenems. Various studies and outbreaks are discussed, highlighting the importance of monitoring and controlling the spread of carbapenem-resistant gram-negative bacteria and other multidrug-resistant organisms in hospital settings. Some studies have developed predictive models and nomograms to identify high-risk patients and personalize risk prediction. Whole-genome sequencing and plasmid characterization techniques have been utilized to analyze the genetic mechanisms of antibiotic resistance and the dissemination of carbapenemases in various bacteria. Increased surveillance and strict infection control measures have been emphasized to contain outbreaks and prevent the further spread of resistance. Overall, the articles demonstrate the urgent need for effective treatment strategies and control measures to combat the growing threat of carbapenem resistance.

The study *“Carbapenem-Resistant Gram-Negative Bacterial Infection in Intensive Care Unit Patients: Antibiotic Resistance Analysis and Predictive Model Development”* analyzed antibiotic resistance in carbapenem-resistant gram-negative bacteria (CR-GNB) in ICU patients and developed a predictive model (Liao et al.). A total of 309 patients with GNB infection were recruited and divided into CR and carbapenem-susceptible (CS) groups. The most prevalent CR-GNB were carbapenem-resistant Klebsiella pneumoniae, carbapenem-resistant Acinetobacter baumannii, and carbapenem-resistant Pseudomonas aeruginosa. A history of combination antibiotic treatments, hospital-acquired infection, and mechanical ventilation ≥ 7 days were independent risk factors for CR-GNB infection. These factors were used to construct a nomogram-based predictive model. The model demonstrated good performance, with an area under the ROC curve (AUC) of 0.753 and 0.718 for the experimental and validation cohort, respectively. The model could be used to guide preventive and treatment measures in identifying patients at high risk of developing CR-GNB infection in the ICU. Another outbreak of multidrug resistant Klebsiella pneumoniae was discussed in the article *“Outbreak of colistin resistant, carbapenemase (blaNDM, blaOXA-232) producing Klebsiella pneumoniae causing blood stream infection among neonates at a tertiary care hospital in India”*, which involved 5 out of 7 neonates (Pathak et al.). The isolates were multidrug resistant, including carbapenems and colistin, and belonged to three different sequence types. The isolates harbored carbapenemase genes and extended-spectrum β-lactamases, but colistin resistance genes could not be detected. K. pneumoniae ST101 was isolated from filtered incubator water and harbored blaNDM-5, blaOXA-232 and ESBL genes, but it was negative for mcr genes. Strict infection control measures were applied, and the outbreak was contained, emphasizing the importance of early detection, surveillance, and proper infection control practices. The study *“Detection of IMP-4 and SFO-1 co-producing ST51 Enterobacter hormaechei clinical isolates”* investigated the genetic characteristics of Enterobacter hormaechei YQ13422hy and YQ13530hy, two multidrug-resistant clinical isolates co-producing the antibiotic resistance genes blaIMP-4 and blaSFO-1 (Qiao et al.). Whole-genome sequencing and plasmid characterization techniques were used to identify and analyze the genes and their genetic context. The study highlights the dissemination of blaIMP-4 in E. hormaechei and the transferable IncN-type plasmid carrying the blaIMP-4 resistance gene in this bacterium. It also emphasizes the importance of understanding the genetic mechanisms of antibiotic resistance for developing effective treatment strategies.

On the other hand, the study *“Development and Validation of Nomograms for Predicting the Risk Probability of Carbapenem Resistance and 28-day All-cause Mortality in Gram-negative Bacteremia among Patients with Hematological Diseases”* aimed to develop two nomograms to predict mortality and carbapenem resistance in hospitalized hematological patients with gram-negative bacteria bloodstream infections (Jian et al.). The study included 244 patients, and 31.6% of them were resistant to carbapenems. The nomograms were constructed using LASSO regression analysis and multivariate logistic regression analysis, and the models were internally validated. The carbapenem resistance nomogram had a modified C-index of 0.788, while the prognosis nomogram had a modified C-index of 0.873. The decision curve analysis demonstrated that the nomograms were clinically practical for predicting high-risk patients. The study suggests that the nomogram models can be an effective tool for personalized risk prediction in clinical practice. Moreover, a carbapenem-resistant Acinetobacter pittii strain, co-producing chromosomal NDM-1 and OXA-820 carbapenemases, was characterized from a bloodstream infection in the manuscript *“Emergence of uncommon KL38-OCL6-ST220 carbapenem resistant Acinetobacter pittii strain, co-producing chromosomal NDM-1 and OXA-820 carbapenemases”* (Tian et al.). The strain was resistant to imipenem, meropenem, and ciprofloxacin but susceptible to amikacin, colistin, and tigecycline. Whole-genome sequencing revealed the strain contained one circular chromosome and four plasmids, with blaNDM-1 and blaOXA-820 genes located in the chromosome. The strain also contained many virulence factors and 12 prophage regions. Phylogenetic analysis showed that the strain was closely related to an A. pittii strain from Anhui, China. Increased surveillance of this species in hospital and community settings is urgently needed due to the challenge presented by the co-existence of these carbapenemases. In this same context of resistance to carbapenems, the study *“Emergence of a Salmonella Rissen ST469 clinical isolate carrying blaNDM-13 in China”* describes a variant of the New Delhi metallo-β-lactamase that confers resistance to carbapenems, which was identified in a patient with fever and diarrhea (Huang et al.). SR33 was found to be multidrug-resistant and carried many virulence genes. The blaNDM-13 was located in a transmissible IncI1 plasmid and had a conserved genetic context and hybrid promoter. This is the first report of blaNDM-13 in Salmonella, highlighting the need for the monitoring and control of its dissemination. IS1294 may be involved in the movement of blaNDM-13.

In terms of transmission, the study *“Genomic transmission analysis of multidrug-resistant Gram-negative bacteria within a newborn unit of a Kenyan Tertiary hospital: a four-month prospective colonization study”* conducted in a Kenyan tertiary hospital investigated the prevalence of multidrug-resistant organisms (MDRO) and carbapenem-resistant organisms (CRO) in a newborn unit using routine microbiology, whole-genome sequencing (WGS), and hospital surveillance data (Villinger et al.). The study included 300 mother–baby pairs and detected MDRO in 16% of neonates at admission, increasing to 44% until discharge, with K. pneumoniae harboring blaNDM-1 and blaNDM-5 being the most frequent CRO. WGS analysis revealed 20 transmission clusters, indicating independent transmission events rather than a large outbreak scenario. The high CRO rate attributed to the spread of NDM-type carbapenemases is a cause for concern.

The prevalence and encoding plasmids in hypervirulent resistant organisms was addressed in two studies: *“* Prevalence of carbapenem-resistant hypervirulent Klebsiella pneumoniae and hypervirulent carbapenem-resistant Klebsiella pneumoniae in China determined via mouse lethality tests *”* (Hu et al.) and *“Hybrid plasmids encoding antimicrobial resistance and virulence traits among hypervirulent Klebsiella pneumoniae ST2096 in India”* (Shankar et al.). The first study investigated the epidemiology of carbapenem-resistant hypervirulent Klebsiella pneumoniae (CR-HvKP) and hypervirulent carbapenem-resistant Klebsiella pneumoniae (Hv-CRKP) in mainland China. The study analyzed 436 Klebsiella pneumoniae strains collected from seven hospitals between 2017 and 2018, using various tests such as sequencing, serotyping, and mouse lethality tests. The study found that the exact prevalence of CR-HvKP is less than 1%, while that of Hv-CRKP is much lower. The authors recommend using mouse lethality tests to accurately determine the prevalence of CR-HvKP and Hv-CRKP.

The second study genetically characterized a collection of multidrug-resistant hypervirulent Klebsiella pneumoniae (MDR-HvKp) ST2096 isolates carrying both antimicrobial resistance (AMR) and virulence genes on a large hybrid plasmid. The hybrid plasmid carried a CRISPR-cas system, which harbored spacer regions against IncF plasmids, preventing their acquisition. This convergence of virulence and AMR in K. pneumoniae is clinically concerning and highlights the continued emergence of such genotypes globally across the species.

Biofilm formation and mortality has been investigated in *“Biofilm formation is not an independent risk factor for mortality in patients with Acinetobacter baumannii bacteremia”* (Chiang et al.). This retrospective study conducted in Taiwan analyzed 711 patients with Acinetobacter baumannii bacteremia, comparing the clinical features of those infected with biofilm-forming and non-biofilm-forming isolates. Multivariate analysis revealed that shock status, higher APACHE II score, lack of appropriate antimicrobial therapy, and carbapenem resistance were independent risk factors for 28-day mortality, but there was no significant difference between the biofilm-forming ability and survival. Patients infected with biofilm-forming isolates had a lower in-hospital mortality rate, and those with congestive heart failure, hematological malignancy, or who received chemotherapy were more likely to be infected with biofilm-forming isolates. Biofilm-forming ability did not influence carbapenem susceptibility.

In Argentina, where Klebsiella pneumoniae and the Enterobacter cloacae complex (ECC) are the two main CRE species, the study *“Novel insights related to the rise of KPC-producing Enterobacter cloacae Complex strains within the nosocomial niche”* shows that the increase in carbapenem-resistant Enterobacterales has led to the urgent need for new antibiotics (Knecht et al.). This study analyzed a patient colonized with both K. pneumoniae and ECC strains and *in vitro* competition assays between high-risk clones of KPC-producing ECC and K. pneumoniae. The results show that these high-risk clones can coexist in the same hospital environment, including the same patient. The findings suggest that the ability of some pandemic clones to compete and occupy a certain niche may explain the worldwide rise of KPC-ECC strains.

## Author contributions

All authors listed have made a substantial, direct, and intellectual contribution to the work and approved it for publication.
